# Tubercle Bacilli in Circulating Blood

**Published:** 1915-10

**Authors:** 


**Tubercle Bacilli in Circulating Blood.** C. R. Austrian and Louis Hamman, Johns Hopkins Hospital Bulletin, August., give an interesting table of the findings of various authorities. Rosenberger, 1909, found bacilli in the blood in all of 300 cases. Kennerknecht, made a similar claim in 1911, for 68 cases by microscopic methods and for 13 cases by animal inoculation. At the other extreme, various series are mentioned in which negative results were obtained, by one or both methods, not always in accordance. The authors failed to find tubercle bacilli in the blood in 50 cases of pulmonary tuberculosis, although inoculation was practiced in 24 cases. Neither were bacilli found in the blood of experimental animals except in late generalized infections or soon after the injection of overwhelming numbers into a vein.

